# Primary Central Nervous System Lymphomas: A Diagnostic Overview of Key Histomorphologic, Immunophenotypic, and Genetic Features

**DOI:** 10.3390/diagnostics10121076

**Published:** 2020-12-11

**Authors:** Marietya I. S. Lauw, Calixto-Hope G. Lucas, Robert S. Ohgami, Kwun Wah Wen

**Affiliations:** 1 Department of Pathology, University of California, San Francisco, CA 94143, USA; Calixto-Hope.Lucas@ucsf.edu (C.-H.G.L.); Robert.Ohgami@ucsf.edu (R.S.O.); Kwun.Wen@ucsf.edu (K.W.W.); 2 Department of Pathology, Helen Diller Family Comprehensive Cancer Center, University of California, San Francisco, CA 94158, USA

**Keywords:** primary central nervous system lymphoma (PCNSL), primary central nervous system diffuse large B-cell lymphoma (PCNS DLBCL), intravascular large B-cell lymphoma, Burkitt lymphoma, dural marginal zone lymphoma (MZL) of mucosa-associated lymphoid tissue (MALT lymphoma), peripheral T-cell lymphoma, NOS (PTCL, NOS), anaplastic large cell lymphoma (ALCL)

## Abstract

Primary central nervous system lymphoma (PCNSL) is a rare form of extranodal non-Hodgkin lymphoma that primarily arises in the brain, spinal cord, leptomeninges, and vitreoretinal compartment of the eye. The term is sometimes used interchangeably with primary central nervous system diffuse large B-cell lymphoma (PCNS DLBCL) because DLBCL comprises a great majority (90–95%) of PCNSL. Although rare, other types of lymphomas can be seen in the central nervous system (CNS), and familiarity with these entities will help their recognition and further workup in order to establish the diagnosis. The latter is especially important in the case of PCNSL where procurement of diagnostic specimen is often challenging and yields scant tissue. In this review, we will discuss the most common types of primary lymphomas that can be seen in the CNS with emphasis on the diagnostic histomorphologic, immunophenotypic, and molecular genetic features. The differential diagnostic approach to these cases and potential pitfalls will also be discussed.

## 1. Introduction

Primary central nervous system lymphoma (PCNSL) is one of a few lymphomas that primarily arise in “immune sanctuary/immune-privileged” sites. Other examples include primary testicular lymphoma and lymphomas in acquired sites of local immune privilege such as breast implants, chronic inflammation, effusions, or other closed spaces within the body [1]. PCNSL is a rare form of extranodal non-Hodgkin lymphoma that primarily arises in the brain, spinal cord, leptomeninges, and vitreoretinal compartment of the eye and shows no significant systemic involvement. Because of its location, procurement of a tissue specimen almost always involves an invasive procedure and, in many cases, only a small amount of sample is obtained. Familiarity with PCNSL and its subtypes will help guide the most optimal approach to maximizing utilization of the limited specimen. This article will discuss PCNSL and its most common subtypes and highlight their diagnostic histomorphologic, immunophenotypic, and molecular features. The differential diagnostic approach to these cases and commonly encountered diagnostic pitfalls will also be discussed. Secondary central nervous system (CNS) involvement by a systemic lymphoma/leukemia will not be discussed as it falls outside the scope of this article. Intravascular large B-cell lymphoma will, however, be briefly discussed here for pathologists to be familiar with this clinically aggressive entity.

The terms ‘PCNSL’ and ‘primary central nervous system diffuse large B-cell lymphoma’ (PCNS DLBCL) have been used interchangeably, as 90–95% of PCNSL are DLBCL. Because of the latter, our next discussion of epidemiologic data, clinical presentation, and diagnostic workup and staging information are most applicable to PCNS DLBCL. The data for other types of PCNSL will be discussed in the following respective subsections.

## 2. Epidemiology

PCNSL comprises 4–7% of all brain tumors [2], 5% of all extranodal lymphomas [3], and less than 1% of all non-Hodgkin lymphomas. It is the third most common malignancy that primarily arises in the central nervous system after glioblastoma and diffuse astrocytoma. In the general population, PCNSL had an annual incidence of 0.43 per 100,000 during the period of 2009–2013 [3]. There is a slight male predominance (ratio of 1.25) and a higher incidence in the Caucasian population in comparison to African American population (ratio of 1.33) [3,4].

**Figure 1:**
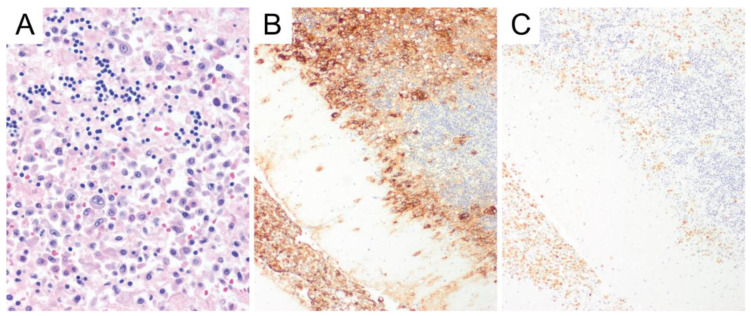
HIV-associated central nervous system lymphoma involving the cerebellum. The tumor cells are large with prominent nucleoli and abundant cytoplasm and are infiltrating between the native Purkinje neurons and internal granular cell layer of the cerebellum (**A**. Hematoxylin and eosin/H&E, original magnification ×400). The tumor cells are positive for both CD20 (**B**, original magnification ×100) and Epstein-Barr virus (EBV) by in situ hybridization (**C**, original magnification ×100).

Two important risk factors of PCNSL are increasing age and human immunodeficiency virus/acquired immunodeficiency syndrome (HIV/AIDS), with a 3600-fold increased incidence of PCNSL ever reported in AIDS patients [4]. Currently, approximately 19% of all patients with PCNSL in United States have HIV [5] (Figure 1). This estimate has been declining since the introduction and widespread use of highly active antiretroviral therapy (HAART) [6]. Whereas the median age of PCNSL at diagnosis is 66 years [3], the diagnosis is usually made at a younger age in HIV/AIDS patients (median of 40.7 years) [7]. Compared to other non-Hodgkin lymphomas, HIV/AIDS patients with PCNSL have the lowest CD4 count (median of 14 cells/µL) at diagnosis [7], reiterating that immunodeficiency is a significant risk factor for developing PCNSL.

## 3. Clinical Presentation

Patients with PCNSL present with varying symptoms according to the central nervous system (CNS) compartment involved. Brain involvement can result in focal neurological deficits, neuropsychiatric symptoms, seizures, and increased intracranial pressure manifesting as headache, nausea, and vomiting [8,9]. Among the aforementioned symptoms, seizure is relatively less common because the cortex is less commonly involved by PCNSL [9]. Spinal involvement by PCNSL usually manifests as discrete intramedullary nodules, and the symptomatology (asymmetric sensory changes, weakness in extremities, and bowel/urinary bladder dysfunction) is similar to that of other intramedullary tumors [9]. Involvement of the peripheral nerve system that includes the peripheral nerves, nerve roots, plexus, and cranial nerves is referred as neurolymphomatosis [10]. It can present with painful peripheral neuropathy or radiculopathy, cranial neuropathy, painless polyneuropathy, and peripheral mononeuropathy or a mononeuropathy multiplex [10]. Ocular involvement by PCNSL, which is seen in 20–25% of cases [11], can result in decreased visual acuity, blurry vision, and/or floaters [9]. Contralateral tumors and parenchymal CNS involvement are seen in a majority of patients (80–90%) with intraocular tumors [12].

## 4. Diagnosis and Staging

An optimal baseline evaluation of patients with PCNSL should include a comprehensive neurological examination, including an assessment of cognitive function, eye examination, and physical examination with special attention to lymph node status and examination of the testes in older men [13]. Eye and testes examinations are of particular importance because the testis and eye are the two sites where PCNSL has the propensity to relapse, with systemic relapse at other sites being much rarer [1,14]. Baseline performance status needs to be carefully documented using Eastern Cooperative Oncology Group (ECOG) performance status and/or Karnofsky performance status because they are widely accepted prognostic variables in addition to age [13].

**Figure 2:**
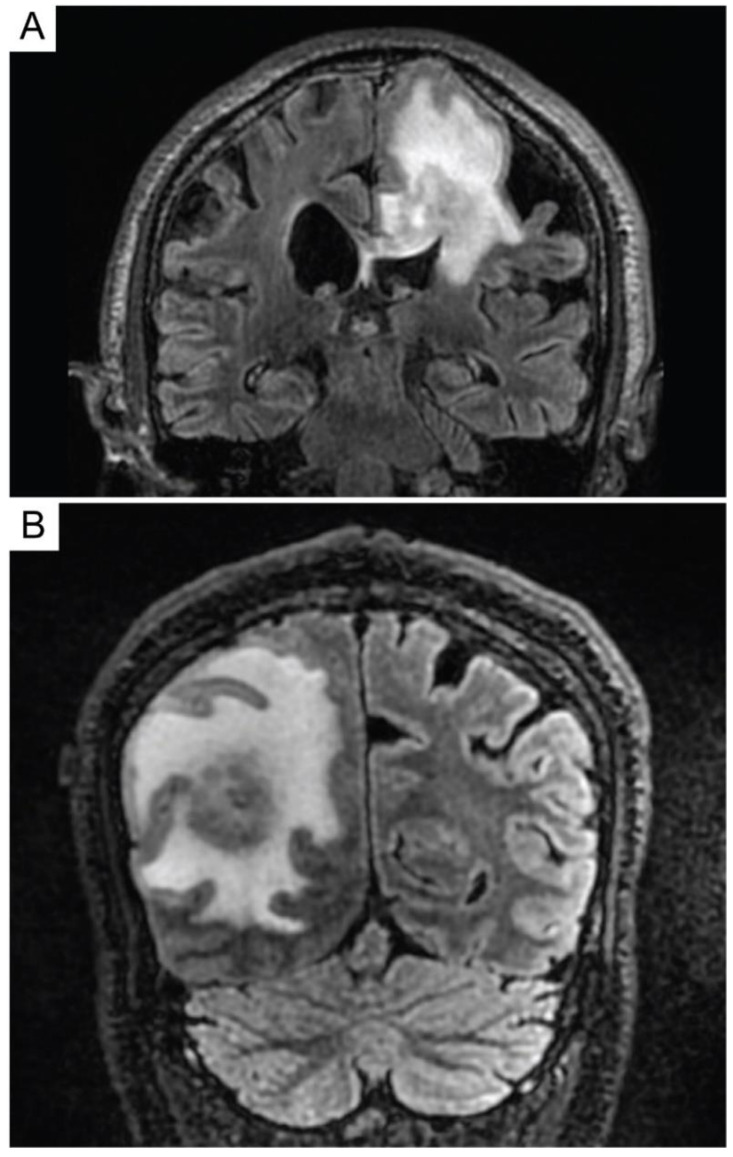
MRI images of a primary central nervous system lymphoma. T2-weighted-Fluid-Attenuated Inversion Recovery (T2-FLAIR) images of patients with primary central nervous system lymphoma that demonstrate T2 hyperintense lesions with increased signal within the surrounding parenchyma. Lesions may mimic primary glial tumors, and when involving the corpus callosum may overlap in appearance with glioblastoma (**A**). Patients may present with a solitary mass with significant peritumoral edema (**B**) or also present with multifocal intracranial disease.

The preferred and most sensitive imaging modality for the evaluation of brain parenchyma in the context of PCNSL is the gadolinium-enhanced magnetic resonance imaging (MRI) scan. Contrast-enhanced computed tomography (CT) scans are an appropriate alternative in patients where MRI scans are contraindicated or when MRI is unavailable [13]. On T1-weighted magnetic resonance (MR) images, PCNSL lesions are hypointense, whereas they are isointense to hyperintense on T2-weighted MR images [9,12] (Figure 2). The amount of peritumoral edema is usually less extensive than in malignant gliomas and metastases [12]. In a retrospective review of 248 cases of primary intracerebral malignant lymphoma by Bataille et al., a majority (66%) of the patients presented with a single lesion, and 90% of the lesions were larger than 1 cm [8]. Eighty-nine percent (175/196) of the lesions analyzed showed supratentorial involvement, and anatomic locations included (in decreasing orders of frequency) the frontal lobe (20%), parietal lobe (18%), temporal lobe (15%), basal ganglia (13%), corpus callosum (11%), brainstem (7%), cerebellum (6%), insula (4%), occipital lobe (4%), and fornix (3%) [8]. A bilateral mirror pattern was seen in 5% of Bataille’s cohort [8].

Tissue biopsy is the diagnostic gold standard in PCNSL. It is of importance that the biopsy is obtained before any corticosteroid administration. Corticosteroid is known to induce apoptosis, resulting in fewer viable tumor cells and a lower sensitivity of the biopsy [15,16]. After corticosteroid therapy, lesional tissue may only show a mixed infiltrate, consisting of macrophages, lymphocytes, plasma cells, and necrosis. The histologic findings of PCNSL after corticosteroid treatment could mimic those of multiple sclerosis (MS) [17]. In the presence of extensive inflammation and patchy and incomplete (rather than confluent and complete) demyelination, the possibility of lymphoma in addition to autoimmune inflammatory demyelination should be considered [17]. In the case of a PCNS B-cell lymphoma that was biopsied after corticosteroid treatment, the necrotic lymphoma cells may still show positivity with an anti-CD20 antibody, which could help to suggest treated B-cell lymphoma [18,19]. However, necrotic cells may also adsorb other lineage markers (including non-hematolymphoid markers), thus precluding a more definite diagnosis and treatment.

## 5. PCNSL in the Pediatric Population

**Figure 3:**
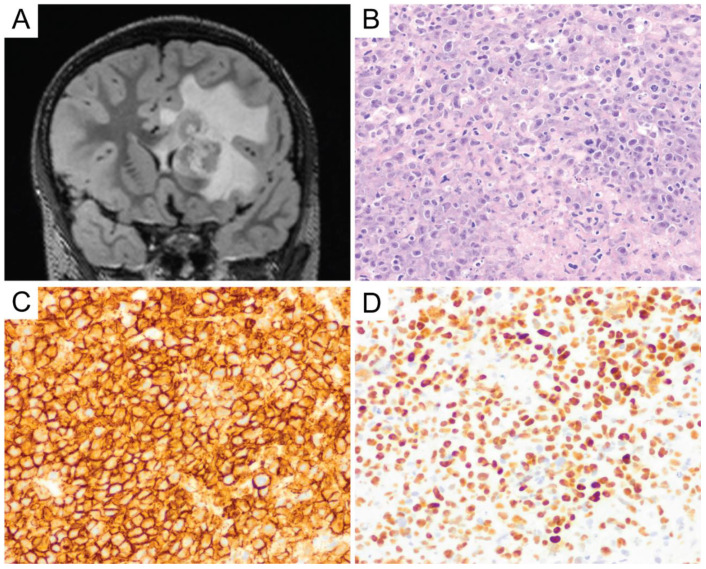
Pediatric primary central nervous system lymphoma (PCNSL). Pediatric PCNSL typically presents as a contrast-enhancing and T2 hyperintense lesion with associated parenchymal edema on MRI (**A**). The tumor cells are large with irregular nuclei and scant cytoplasm, often with associated necrosis (**B**, original magnification ×400). The tumor cells are positive for CD20 (**C**, original magnification ×400). The lymphoma cells in this example were of germinal center phenotype, showing no expression of CD10 (not shown) and MUM1 (not shown), and were positive for Bcl-6 (**D**, original magnification ×400).

In the pediatric setting (0–19 years), PCNSL is very rare, comprising approximately 1% of all PCNSL cases [20], with an incidence of 0.01 per 100,000 in the period of 2009–2013 [3]. It affects males more than females (male-to-female ratio of 1.7:1) and the median age of diagnosis is 12.5–14 years [21,22]. Children with immunodeficiency, either congenital or acquired, are at highest risk of developing PCNSL, although most cases of pediatric PCNSL in the last decades were reported in immunocompetent children [3,22]. Similar to adults, diffuse large B-cell lymphoma (DLBCL) accounts for the majority (49–70%) of PCNSL in the pediatric population (Figure 3B–D), followed by anaplastic large cell lymphoma (17–23%), Burkitt lymphoma (7–12%) and lymphoblastic lymphoma (7%) [22,23].

Children with PCNSL usually present with symptoms of increased intracranial pressure, such as headaches/nausea/vomiting, double vision, facial nerve palsy, dysarthria, ataxia, and loss of consciousness [21]. On imaging, the tumor can be solitary or multifocal, and the most common locations of the tumor are the parietal and frontal lobes, followed by the cerebellum, pituitary stalk, hypothalamus, and temporal lobe [21] (Figure 3A). Unlike PCNSL in adults (discussed below), the genetic landscape of pediatric cases is largely unknown. Our unpublished data suggest that pediatric PCNSL cases do not harbor the common genetic alterations (e.g. *MYD88*, *CARD11*, *CD79B*, *PIM1*) seen in their adult counterparts but are enriched with *TP53* mutations. Its rarity and the lack of data from large prospective trials pose a challenge to determine the most appropriate treatment for pediatric PCNSL patients. However, a recent study by Attarbaschi et al. showed that histological subtype-driven therapy, which included high-dose methotrexate, high-dose cytarabine, steroids, and no irradiation (except for anaplastic large cell lymphoma), resulted in a good outcome [22]. Their study reported a 5-year event-free survival (EFS) and overall survival (OS) of 74% ± 5% and 85% ± 4%, respectively [22]. These were in line with the previously reported numbers, 2-year progression-free survival (PFS) and overall survival (OS) rates of 61% and 86%, respectively [21]. PCNSL in children appears to have a better prognosis than in adults, and the better prognosis could be due to the higher tolerance of very young patients to higher doses of methotrexate and different biology of the lymphomas [21]. In their study, Attarbaschi et al. found that preexisting disorders were associated with inferior EFS and OS, and that the usage of high-dose methotrexate, high-dose cytarabine, and alkylators was associated with improved prognosis [22].

## 6. Primary Central Nervous System Diffuse Large B-Cell Lymphoma (PCNS DLBCL)

Diffuse large B-cell lymphoma (DLBCL) accounts for 90–95% of all PCNSL [5]. PCNS DLBCL includes DLBCL arising within the brain, spinal cord, leptomeninges, and vitreo-retina [12]. DLBCL of the dura and ocular adnexa, intravascular large B-cell lymphomas, those of systemic disease (secondary lymphomas), and most immunodeficiency-associated lymphomas (e.g., in the posttransplant or iatrogenic-related setting) are excluded from this category [12]. The etiology of PCNS DLBCL in the immunocompetent population is still unknown. Viruses such as Epstein-Barr virus (EBV), human herpesvirus-6 (HHV-6) [24], human herpesvirus-8 (HHV-8) [25], and polyomaviruses [26,27] have been shown to play no causative role.

**Figure 4:**
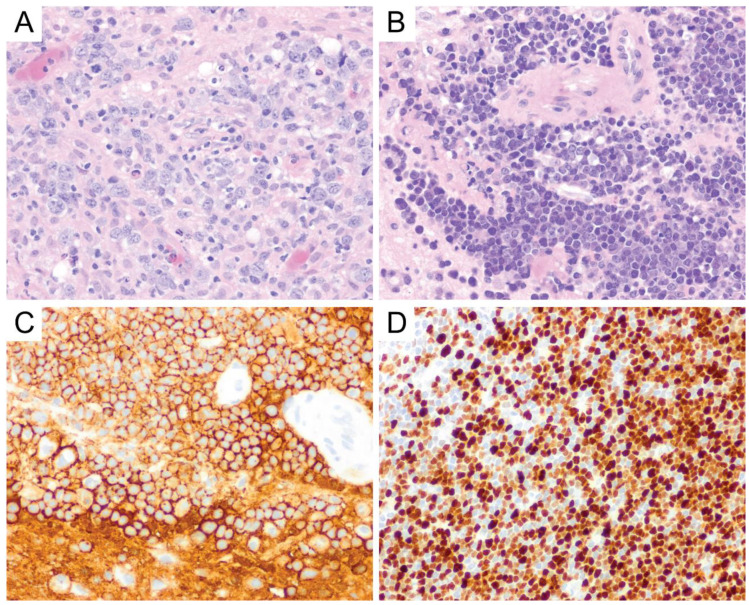
Primary CNS DLBCL. Lymphoma cells are round to oval and contain irregular, vesicular nuclei with prominent nucleoli, morphologically consistent with centroblasts or immunoblasts (**A**, original magnification ×400). Primary CNS DLBCL usually shows perivascular arrangement (angiocentricity) with tumor cells forming layers around the blood vessels (**B**, original magnification ×400). They express CD20 (**C**, original magnification ×400) and usually show a high Ki-67 proliferation index (**D**, original magnification ×400).

On gross/macroscopic examination, PCNS DLBCL shows a soft and pale, fish flesh appearance. It is characterized histomorphologically by a diffuse proliferation of medium-to-large-sized lymphoid cells with pleomorphic, round to oval, irregular, and vesicular nuclei with prominent nucleoli, morphologically consistent with centroblasts or immunoblasts (Figure 4A). Tumor cells usually exhibit perivascular arrangement (angiocentricity) by forming layers around blood vessels (Figure 4B). Similar to high-grade glial neoplasms, areas of geographic necrosis are commonly present in the center of the tumor. However, microvascular proliferation (frequently encountered in high-grade gliomas) is rare in primary CNS DLBCL and is often nonexistent [12]. A biopsy obtained from the periphery of the tumor could pose a diagnostic challenge, because the lymphomatous cells can infiltrate the brain parenchyma as single cells and can be easily obscured by the background astrogliosis and reactive inflammatory cells consisting of T-cells, B-cells, and foamy histiocytes [12]. Immunohistochemical stains for lymphoid markers should be performed if there is any suspicion for lymphoma. The presence of reactive perivascular T-cell infiltrate, defined as the presence of at least one small-to-medium-sized vessel surrounded by a rim of small-to-intermediate-sized T-cells, may correlate with favorable outcomes and should be described in the report when observed [28].

PCNS DLBCL is a mature B-cell neoplasm, and the tumor cells express B-cell markers, particularly CD19, CD20 (Figure 4C), CD22, CD79a, and PAX5 [12]. A majority (67–96%) [29,30,31,32] of cases are of an activated B-cell (ABC)/non-germinal center B-cell (non-GCB) subtype where the tumor cells are either (i) CD10-negative, Bcl-6-negative or (ii) CD10-negative, Bcl-6-positive, and MUM1-positive by immunohistochemistry using the Hans algorithm [33]. The remainder of the cases are of a germinal center B-cell (GCB) subtype, which is defined by CD10 expression in at least 30% of tumor cells or Bcl-6 expression in the absence of MUM1 expression [33]. Bcl-6 and MUM1 are expressed in the majority of PCNS DLBCL, but CD10 expression is only seen in a small portion of cases (<10%) [1,12]. Therefore, CD10 expression should always prompt the investigation of a potential systemic DLBCL that has disseminated into the central nervous system. In a study by Montesinos-Rongen et al., reverse transcriptase-polymerase chain reaction (RT-PCR) for transcripts of immunoglobulin constant region gene segments, performed on 11 PCNS DLBCL samples, showed exclusive transcription of IgM and IgD mRNA in the absence of IgG, IgA, or IgE transcription [34]. Neoplastic Bcells can show either kappa or lambda light chain restriction, but plasma cell markers (i.e., CD138 and CD38) are usually negative [12]. The Ki-67 proliferation index in PCNS DLBCL is usually very high, up to 90% [35] (Figure 4D), and it was suggested that the high proliferation index might correlate well with the frequently observed c-Myc expression in PCNS DLBCL. This hypothesis was supported by the observation that, in Brunn’s series, two of the three cases of primary CNS DLBCL with a relatively low Ki-67 proliferation index (i.e., <50%) also showed low c-Myc expression [35]. EBV expression in PCNS DLBCL is not common and may reflect an underlying immunodeficiency [12].

The worse prognosis of PCNS DLBCL in comparison to systemic DLBCL was first thought to be due to the significant portion of cases with an ABC subtype [30], which is generally associated with a poorer prognosis in comparison to the GCB subtype. However, some studies reported no significant difference in the overall survival and progression-free survival between the ABC and GCB subtypes of PCNS DLBCL [29,36]. Interphase cytogenetic analysis showed that *IGH* and *BCL6* rearrangements were seen in, respectively, 13% and 17–26% of PCNS DLBCL [35,37,38]. Whereas systemic DLBCL with co-expression of c-Myc and Bcl-2 (double-expressor) and/or co-rearrangement of *MYC* and *BCL2* and/or *BCL6* (double or triple hit lymphoma) has been associated with a worse prognosis, the findings were inconclusive for PCNS DLBCL. The expression of c-Myc and Bcl-2 proteins has been described in a significant portion of PCNS DLBCL, respectively, up to 70–90% [35,36] and 59–73% [39,40,41] of cases. Interestingly, *MYC* gene rearrangements are significantly lower in frequency (3–8%) [35,37], and thus far, *BCL2* gene rearrangements have not been reported [35,42] in PCNS DLBCL. This suggests that increased c-Myc and Bcl-2 protein expressions might be attributable to other genetic aberrations, such as *MYC* and *BCL2* gene mutations or altered regulation of expression (e.g., through post-transcriptional and post-translational modifications) independent of *MYC* or *BCL2* gene rearrangements. Although some studies showed that positive c-Myc protein expression (defined as positive immunohistochemical staining in at least 40% of tumor cells) was associated with worse overall and progression-free survival [39,40,43], other studies found no significant difference in prognosis [29,36]. It is important to note that these studies used the same cutoff to define positive c-Myc protein expression. Tapia et al. used a threshold greater than 30% to define c-Myc positivity and reported the association of c-Myc expression with lower overall survival [41]. It is still unclear why these results have varied across the studies and it does not seem to be related to the c-Myc antibodies used. It needs to be noted that Kim’s group used c-Myc antibodies from Cell Marque and they reported a significantly lower c-Myc-positive cases (18.1%) in their cohort [40]. Similarly, inconclusive findings were observed for Bcl-2 protein expression. Shi et al. [39], Kim at al. [40], and Makino et al. [29] reported a less favorable overall survival associated with high Bcl-2 protein expression, while Tapia et al. [41] and Liu et al. [44] reported no significant difference in prognosis. It is unclear whether the inconclusive findings across the Bcl-2 studies were caused by different cutoffs used to define positivity for Bcl-2, which ranged from 50–70% [29,39,40,44]. However, Tapia et al. evaluated several cutoffs to define Bcl-2 positivity, 30%, 50%, and 70%, and found no significant association between Bcl-2 expression and prognosis [41]. Similarly, several studies reported a more adverse clinical outcome associated with co-expression of c-Myc and Bcl-2 [40,45] and other studies failed to demonstrate such an association [29,36]. Given these inconclusive data, the decision to perform immunohistochemistry for cell-of-origin status (i.e., GCB vs. ABC), double expressor status, and/or fluorescence in situ hybridization for *MYC*, *BCL2*, and *BCL6* rearrangements may be highly variable in various practices and will at least be partly based upon the preference of the treating oncologists.

The advent of genetic analysis has expanded the knowledge in the histogenetic origin of PCNS DLBCL. For example, the presence of mutations in the 5’-noncoding region of the *BCL6* gene (a marker of B-cell transition through the germinal center) in a significant subset of PCNS lymphomas suggests that this lymphoma might be derived from germinal center Bcells. Montesinos-Rongen et al. further showed that all PCNS DLBCLs in their series carried monoclonally rearranged V region genes and had introduced somatic mutations into the rearranged *IG* genes [46]. These findings suggest that PCNS DLBCLs are derived from mature B-cells with prior antigenic exposure and have undergone T-cell-dependent affinity maturation in the microenvironment of the germinal center of secondary lymphatic organs [46].

Despite their histomorphologic and immunophenotypic resemblance to systemic DLBCL, PCNS DLBCL might represent a distinct subtype that is characterized by their unique clinical and molecular features related to the CNS milieu. The immunologically privileged microenvironment of the CNS has been one of the subjects in the earlier studies of pathogenesis of PCNSL, and it was postulated that an interplay between chemokines and chemokine receptors or cytokines within the CNS microenvironment might play a key role in the development of PCNSL [12,47]. Interleukin (IL)-4, a B-cell growth and survival factor, has been found to be expressed by tumor cells and tumor vasculature in CNS lymphomas [48]. Several IL-4-induced gene products, including X-box binding protein 1 (XBP-1), have been found to be highly expressed in PCNSL [48]. XBP-1 regulates unfolded protein response (UPR) and both UPR and XBP-1 are essential for tumor growth under hypoxic stress and glucose deprivation [48]. In addition, the activated form of signal transducer and activator of transcription 6 (STAT6), a mediator of IL-4 signaling, was expressed by tumor cells and tumor endothelia in PCNSL, and a high expression of activated STAT6 was associated with shorter survival in patients with PCNSL who were treated with high-dose intravenous methotrexate therapy [48].

**Figure 5:**
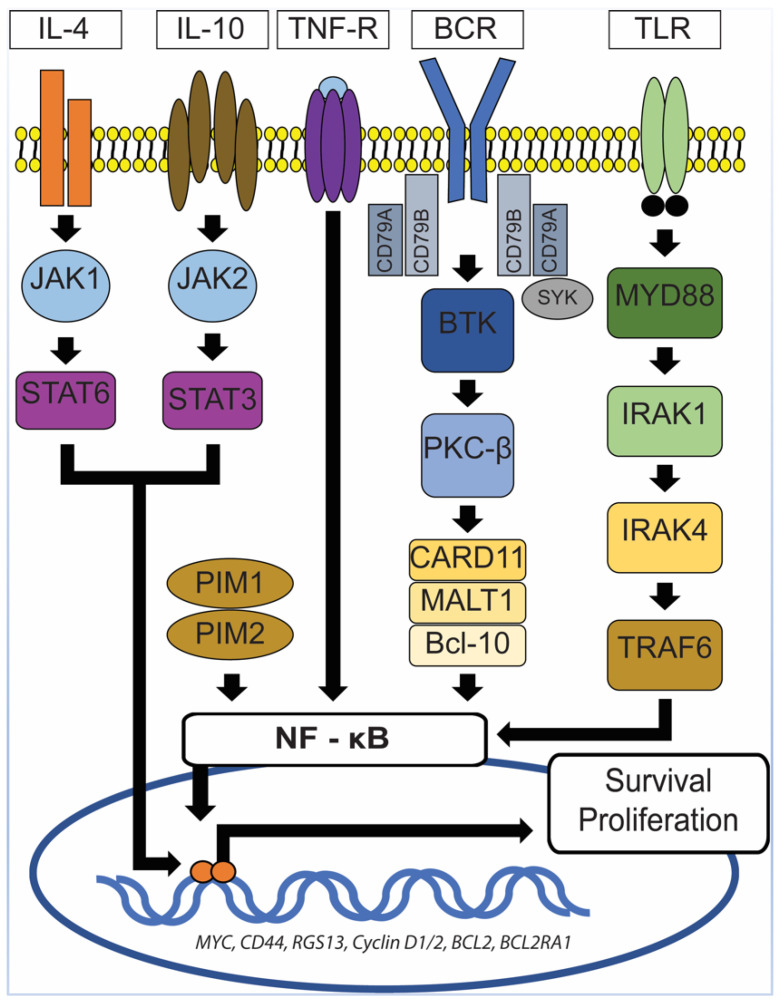
NF-ĸB signaling pathway. In PCNSL, activation of NF-ĸB signaling pathway can be mediated through the gain of 18q21.33-q23, activating the mutation of CARD11 and stimulation of the B-cell receptor (BCR), tumor necrosis factor (TNF), or toll-like receptor (TLR) pathway. MYD88 mutations, which are frequently seen in PCNSL, lead to spontaneous assembly of a protein complex containing IRAK1 and IRAK4, ultimately resulting in NF-ĸB signaling pathway activation, Janus kinase (JAK) activation of STAT3 pathway, and the secretion of IL-6, IL-10, and interferon-β. TNF-R: TNF receptor, MYD88: Myeloid differentiation primary response gene 88, CARD11: Caspase recruitment domain family member 11, IRAK1/4: Interleukin 1 receptor-associated kinase 1/4, TRAF6: Tumor necrosis factor receptor (TNFR)-associated factor 6, BCR: B-cell receptor, SyK: Spleen tyrosine kinase, BTK: Bruton’s tyrosine kinase, PKC-β: Protein kinase C β type, MALT1: Mucosa-associated lymphoid tissue lymphoma 1, Bcl-10: B-cell lymphoma/leukemia 10, JAK1/2: Janus kinase 1/2, STAT3/6: Signal transducer and activator of transcription 3/6, IL-4/10: Interleukin 4/10.

Aberrant and constitutive activation of nuclear factor (NF)-ĸB signaling pathway is a hallmark of PCNSL (Figure 5), which can be mediated through the gain of 18q21.33-q23, activating mutation of *CARD11* (caspase recruitment domain family member 11) and stimulation of the B-cell receptor (BCR), tumor necrosis factor (TNF), or toll-like receptor (TLR) pathway [49]. The gain of 18q21.33-q23 has been described in 43% of PCNSL and is one of the most common chromosomal abnormalities seen in PCNSL [50]. Other commonly seen chromosomal abnormalities include the loss of 6q21 (52%) and 6p21.32 (37%) and gain of 19q13.43 (47%), 1q, 7, and 12 [50]. The *CARD11* gene encodes for the CARD11 protein, a mediator of the BCR pathway [49]. The TLR pathway deregulation in PCNSL is commonly secondary to mutations in the myeloid differentiation primary response gene 88 (*MYD88*), which is highly recurrent in PCNSL and has been described in at least 50% of PCNSL [12,42,49]. This is at a higher rate than described for systemic DLBCL (10–20%) [42]. MYD88 is an adaptor protein that mediates TLR and IL-1 receptor signaling [51]. It associates with interleukin 1 receptor-associated kinase 1 (IRAK1) and IRAK4 to promote tumor survival [51]. In 71% of PCNSL with *MYD88* mutations, a leucine to proline exchange at position 265 (L265P) has been found [49]. *MYD88* L265P is an activating somatic mutation that promotes survival by spontaneously assembling a protein complex containing IRAK1 and IRAK4, ultimately leading to NF-ĸB signaling pathway activation, Janus kinase (JAK) activation of STAT3, and the secretion of IL-6, IL-10, and interferon-β [49,51]. IL-10 may play a pivotal role in suppressing immune reactions toward tumor cells, hence promoting their survival. Using whole-exome sequencing of 41 PCNS DLBCL cases, Fukumura et al. identified other frequently mutated genes in the NF-ĸB signaling pathway, including *PIM1*, *BTG2*, *CD44*, *XBP1*, *CD79B*, and *NFKB1E* [42]. Their study also showed that mutations in *PIM1* (100%) and *BTG2* (92.7%) were more frequently seen in PCNS DLBCL in comparison to systemic DLBCL [42]. In contrast, *RHOH* and *BCL6*, which are frequent targets of aberrant somatic hypermutation in systemic DLBCL, were not affected in PCNS DLBCL [42]. These findings lend further support for the notion that PCNS DLBCL represents a distinct entity from systemic DLBCL. In the same study, Fukumura et al. also examined prognostic values and potential therapeutic implications of their results. They demonstrated that alterations in *HLA-C* were associated with a shorter progression-free survival (PFS) and that activation of the genes at the 7q35 locus might contribute to PCNS DLBCL relapse [42]. In addition, chromosome copy number alterations involving *TP53* were not associated with a shorter progression-free survival [42]. Furthermore, 6 of the 41 cases in the Fukumura’s series carried *GRB2* mutations, a gene that codes for an adapter protein that binds to tyrosine kinases and other docking proteins through its Src homology 2 (SH2) domain and transduces growth signals through the RAS-MAPK pathway [42]. When GRB2(V140G)-expressing 3T3 cells were treated in vitro with MAP2K1/2 inhibitors (trametinib and selumetinib), their malignant transformation was attenuated [42], a promising result that merits further investigations.

The main differential diagnosis in a case of PCNS DLBCL includes secondary involvement by systemic DLBCL, Burkitt lymphoma (BL, primary versus secondary, will be discussed below), and high-grade B-cell lymphoma (HGBL, a terminology used in the revised fourth edition of WHO Classification of Tumours of Haematopoietic and Lymphoid Tissues) [12]. Two categories of HGBL have been described: HGBL with *MYC* and *BCL2* and/or *BCL6* rearrangements, and HGBL, not otherwise specified (NOS) [12]. Because, to our knowledge, *BCL2* rearrangement has not been reported in PCNS DLBCL [35,42], the type of HGBL that will be encountered in PCNSL will most likely be HGBL, NOS, or HGBL with *MYC* and *BCL6* rearrangements. HGBL, NOS is a heterogenous group of mature B-cell lymphomas that share morphologic, immunophenotypic, and genetic features with DLBCL and BL [12]. Whereas they may have *MYC* gene rearrangements, they are negative for both *MYC* plus *BCL2* and/or *BCL6* rearrangements [12]. Large, mature B-cell lymphomas with blastoid (medium-sized cells with fine, powdery chromatin) morphology fall into this category (HGBL, NOS) [12]. They can resemble Burkitt lymphoma in morphology, displaying sheets of monomorphic cells with scattered mitotic figures and apoptotic debris, intermixed with tingible body macrophages and imparting a starry sky appearance, but show immunophenotype and molecular genetic findings that are incompatible with BL [12]. In the cases with blastoid morphology, two main differential diagnoses need to be ruled out, namely B-lymphoblastic lymphoma/leukemia (positive for TdT and/or CD34) and the blastoid variant of mantle cell lymphoma (positive for Bcl-1/cyclin D1 and/or SOX11). HGBL is a rare and relatively new entity and there is paucity of data regarding its behavior in the PCNSL setting. Whereas a hematopathologist may make an effort to distinguish DLBCL and HGBL entities and to derive a more precise diagnosis, the therapeutic implications could be highly variable depending upon the preference of treating oncologist and specimen/resource availability.

## 7. Intravascular Large B-Cell Lymphoma

Even though it is rarely isolated to the CNS and hence does not qualify as a PCNSL, intravascular large B-cell lymphoma is described here because of its frequent CNS involvement and familiarity to this entity will allow an early diagnosis and treatment of this aggressive lymphoma. Intravascular large B-cell lymphoma is an extranodal large B-cell lymphoma that typically presents in older adults and usually follows an aggressive clinical course, with many patients diagnosed at perimortem or on postmortem examination. Approximately one-third of the patients present with neurologic symptoms. Clinically, two patterns of presentations have been described: The classical variant that is historically referred to as the Western variant and the hemophagocytic syndrome-associated variant that is historically referred to as the Asian variant [12]. The classical variant typically presents with skin and CNS involvement [12,52]. In contrast, the hemophagocytic syndrome-associated variant often involves multiple organs, including the CNS, disseminates widely at time of diagnosis, and can be associated with hemophagocytic lymphohistiocytosis (HLH, which may overlap with the clinical presentation of other CNS lymphomas) [12,52,53]. Nodal involvement is rare in either variant [12]. Patients with CNS involvement can present with rapidly progressive cognitive dysfunction or subacute dementia [54]. Other nonspecific symptoms include fever, anemia, and thrombocytopenia. Radiographically, there are no discernable mass lesions, but FLAIR or T2 weighted abnormalities may be seen [55]. Imaging abnormalities are typically present predominantly within the periventricular white matter, raising the differential of a leukoencephalopathy. Standard treatment includes rituximab along with other large B-cell lymphoma and CNS-directed chemotherapy regimens. The overall prognosis is poor, with a median survival of 5–7 months in some case series. However, recent data suggests a 50% 5-year survival when treatment can be initiated early on in the disease course [56].

**Figure 6:**
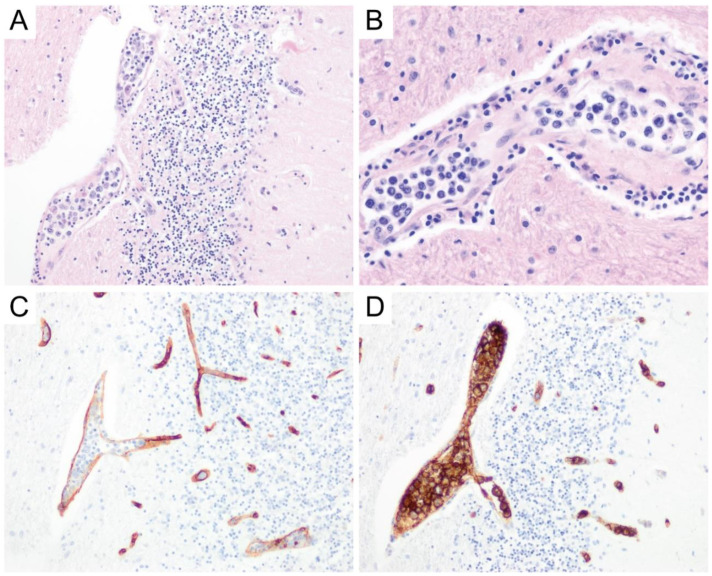
Intravascular large B-cell lymphoma. Intravascular large B-cell lymphoma demonstrates a proliferation of tumor cells confined to the lumina of blood vessels (**A**, original magnification ×200). Tumor cells have large round nuclei with prominent nucleoli and scant cytoplasm (**B**, original magnification ×400). Immunohistochemically, the endothelial cells of blood vessels can be highlighted using antibodies against CD34 (**C**, original magnification ×200). The neoplastic lymphocytes within vessels express CD20, confirming B-cell lineage (**D**, original magnification ×200).

On postmortem examination, the brain may appear grossly normal. On occasion, there may be dusky gray discoloration of white matter with focal lesions resembling infarcts. Microscopically, neoplastic lymphoid cells can be seen filling the lumina of small- or intermediate-sized vessels within the brain parenchyma or leptomeninges [12] (Figure 6A), but such vascular findings may be very focal and subtle to identify. The neoplastic lymphocytes typically have large round nuclei with prominent nucleoli and scant cytoplasm [12] (Figure 6B). Mitotic figures are often easily identified [12]. The involvement of vessels can be demonstrated using CD34 immunohistochemistry that highlights endothelial cells (Figure 6C). There are often no associated changes in the brain parenchyma, but changes related to infarction, including intravascular fibrin thrombi, necrosis of vessel walls, and surrounding parenchymal gliosis, may be seen. The involvement of multiple vessels by neoplastic B-cells is sufficient for the diagnosis of intravascular large B-cell lymphoma. These cells can be highlighted immunohistochemically by CD45, CD20 (Figure 6D), PAX-5, and CD79a [55]. CD5 and CD10 co-expression can be seen in a subset of this lymphoma (in 38% and 13%, respectively) and almost all cases that show negative expression of CD10, are MUM1-positive [12]. Interestingly, they lack CD29 (Integrin beta-1) and CD54 (ICAM-1), which are adhesions molecules necessary for lymphocyte trafficking and transvascular migration, correlating with the intravascular confinement of neoplastic lymphocytes with absence of parenchymal involvement in the majority of cases [57]. Mutational analysis revealed recurrent alterations in *MYD88* and *CD79B* in intravascular large B-cell lymphoma that are commonly mutated in other types of PCNS DLBCL [58].

The main differential diagnosis for intravascular large B-cell lymphoma includes intravascular natural killer cell (NK) lymphomas, intravascular T-cell lymphomas, and intralymphatic ALK-negative anaplastic large cell lymphomas (ALCL) [12]. Immunohistochemical stains for T-cell markers to include CD30, NK-cell markers, and B-cell markers would help distinguish the three differential diagnoses.

## 8. Burkitt Lymphoma

Primary CNS Burkitt lymphoma (PCNSBL) is extremely rare and the exact incidence is unknown. Thirty-eight cases of PCNSBL have been reported in the literature to date [59,60]. PCNSBL has been described in all age groups, ranging from 6 months old to 75 years old, and has been described as an intracranial as well as spinal mass [59]. There is male predominance (~2:1) [59,60].

Grossly, the mass is firm/elastic and grey-white [61]. Histologically, PCNSBL is composed of diffuse proliferation of monotonous medium-sized lymphocytes with round nuclei, finely clumped chromatin, multiple paracentral nucleoli, and scant basophilic cytoplasm [12]. The cytoplasm usually contains lipid vacuoles that are more easily identified on touch or smear preparations [12]. Perivascular cuffing by tumor cells has also been described [61]. Mitoses and apoptotic bodies are easily identified with scattered tingible body macrophages, giving it a starry sky appearance. The lymphoma cells show a similar immunohistochemical profile to Burkitt lymphoma outside of the CNS. They express B-cell antigens that include CD19, CD20, CD22, CD79a, and PAX5, and show a germinal center phenotype (CD10 and Bcl-6) with moderate to strong membrane IgM with light chain restriction [12]. Similar to their systemic counterpart, strong c-Myc expression is usually observed in the tumor cells and the Ki-67 proliferation index usually approaches 100% [12]. Tumor cells are usually negative for CD5, CD23, CD138, Bcl-2, and TdT [12]. At the cytogenetic level, Burkitt lymphoma is hallmarked by the translocation between *MYC* (8q24) and *IGH* (14q32) or less commonly *IGK* (2p12) or *IGL* (22q11) [12]. Nevertheless, approximately 10% of Burkitt lymphoma can lack an identifiable *MYC* rearrangement, and in the presence of strong c-Myc expression by immunohistochemistry, it suggests an alternative mechanism of *MYC* gene deregulations [62]. Next-generation sequencing has revealed recurrent mutations in the transcription factor *TCF3* or *E2A* and its negative regulator *ID3* in approximately 70% of sporadic BL cases [63,64,65]. Recurrent mutations have also been reported in *CCND3*, *TP53*, *RHOA*, *SMARCA4*, and *ARID1A* [63,64,65,66,67].

The differential diagnosis includes high-grade B-cell lymphoma, anaplastic astrocytoma, metastatic small cell carcinoma, and melanoma. Fluorescence in situ hybridization (FISH) study for *MYC*, *BCL2*, and *BCL6* rearrangements should be performed on every Burkitt lymphoma case, because the presence of *MYC* and *BCL2* and/or *BCL6* in what is otherwise a Burkitt lymphoma makes it a high-grade B-cell lymphoma per the current edition of WHO Classification of Tumours of Hematopoietic and Lymphoid Tissues [12].

## 9. Low-Grade B-Cell Lymphomas and T-Cell Lymphomas

In contrast to DLBCL, little is known about primary low-grade B-cell lymphomas and T-cell lymphomas in the CNS. The paucity of data is mostly due to the rarity of these entities. The largest series to date was reported by Jahnke et al., which included 40 patients with low-grade PCNSL diagnosed from 1979 to 2004 [68]. In their series, 80% of the patients had B-cell lymphomas, with the rest having T-cell lymphomas [68]. The B-cell lymphomas consisted of 34% (11/32) lymphoplasmacytic lymphoma (LPL), 3% (1/32) grade 1 follicular lymphoma (FL), and 62.5% (20/32) small lymphocytic lymphomas that were not further classified [68]. It is unclear whether a subset of the “lymphoplasmacytic lymphoma” cases in these series would still qualify as LPL or as marginal zone lymphoma now that *MYD88* genetic testing has become available. In Jahnke’s series, the median age of presentation was 60 years [68]. The tumor was mostly supratentorial and the involvement of a cerebral hemisphere or deeper brain structures was present in 92.5% of patients [68]. Evidence of leptomeningeal involvement was seen in 10% of patients, and only 1 out of 40 patients had an isolated spinal cord disease [68]. MRI showed a single lesion in 65% (11/17) of the cases and 2 lesions in 12% (2/17) of cases, with the rest showing more than 2 lesions [68]. Some features on the MRI that are usually not seen in PCNS DLBCL include hyperintensity on T2-weighted images, inhomogeneous contrast enhancement, moderate or absent contrast enhancement, and lack of periventricular localization [68].

Establishing a precise diagnosis in low-grade lymphomas is relatively more challenging than in PCNS DLBCL because it usually requires a broader panel of immunohistochemical stains. This could pose a challenge when the biopsy specimen is of limited nature. Therefore, appropriate allocation of fresh biopsy specimen for different ancillary studies is of utmost importance. When the findings on the smear preparation and/or frozen section raise a suspicion of low-grade lymphomas, a portion of the specimen should be sent for flow cytometry. Primary CNS low-grade B-cell lymphomas, like PCNS DLBCL, reportedly also show predominantly perivascular lymphocytic infiltrates and frequent lymphoplasmacytic differentiation [69,70]. A basic panel of immunohistochemical stains for a suspected small B-cell lymphoma would include at least CD20, PAX5, CD3, CD5, CD23, CD10, Bcl-6, Bcl-2, CD43, Bcl-1, SOX11, EBV in situ hybridization (ISH), and Ki-67, with addition of kappa and lambda immunostains and/or in situ hybridization studies in the presence of plasma cells. Immunohistochemical stains for a suspected T-cell lymphoma would include CD3, CD4, CD8, CD2, CD5, CD7, CD56, CD30, and EBV ISH. Performing immunohistochemical stains in stages could be considered in the case of limited samples. Cytotoxic markers (TIA-1, granzyme B, perforin) and T-follicular helper markers (PD-1, CD10, Bcl-6, CXCL13, ICOS) could be added for further subclassification.

Although the exact frequency of low-grade B-cell lymphoma subtypes in CNS is not known, the majority seem to be comprised of small B-cell lymphomas with plasmacytic differentiation with differential diagnosis, including marginal zone lymphoma (MZL) and lymphoplasmacytic lymphoma (LPL) [68,69,70]. MZL and LPL share similar histomorphologic findings, with lymphoplasmacytic infiltrate composed of small-to-medium-sized lymphocytes with slightly irregular nuclei, mature chromatin, inconspicuous nucleoli, and relatively abundant pale cytoplasm [12]. The lymphocytes can have a monocytoid morphology due to the abundant pale cytoplasm and they are admixed with variable numbers of plasma cells. In the five MZL cases in Nomani’s series, the infiltrate was perivascular, and in one case, reactive germinal centers were seen [70]. The diagnosis of MZL and LPL require exclusion of follicular lymphoma (by negative expression of CD10, Bcl-6, and FMC7), chronic lymphocytic leukemia (CLL)/small lymphocytic lymphoma (SLL, by negative expression of CD5, CD23, LEF1, and CD200), and mantle cell lymphoma (MCL, by negative expression of CD5, Bcl-1/cyclin D1, and SOX11). The clonal nature of the B-cells in MZL and LPL can be established by immunohistochemical stains and/or in situ hybridization studies for kappa and lambda light chains and is usually detected by flow cytometry. The distinction between MZL and LPL can be challenging and needs correlation with other clinical and laboratory findings (i.e., presence/absence of lymphadenopathy, hepatosplenomegaly, bone marrow involvement, and M-protein). The presence of *MYD88* L265P mutation favors LPL above MZL because this mutation is seen more commonly in LPL than in MZL (>90% in LPL versus ~10% in MZL) [71,72]. Two of the four parenchymal MZL cases in Nomani’s series, in which polymerase chain reaction (PCR) for *MYD88* L265P was performed, were negative for the mutation [70]. Evidence of systemic involvement (i.e., lymphadenopathy, hepatosplenomegaly, and bone marrow involvement) in a case that would qualify as LPL should warrant a diagnosis of CNS involvement by LPL (Bing–Neel syndrome) and not primary CNS LPL. It needs to be noted that parenchymal MZL may have a less favorable prognosis in comparison to dural marginal zone lymphoma (described below), which might partly due to the better resectability of the latter [70].

## 10. Dural Marginal Zone Lymphoma (MZL)

This entity is described separately because of the frequent involvement of the meninges by primary as well as secondary low-grade B-cell non-Hodgkin lymphomas (NHL) [73]. Within low-grade B-cell NHL, marginal zone lymphoma is the most common subtype that is encountered in dural lymphomas. Dural marginal zone lymphoma is thought to fall in the spectrum of MALT (mucosa-associated lymphoid tissue) lymphomas due to the similar indolent clinical behavior, favorable response to treatment, and morphologic and immunophenotypic features [73,74].

**Figure 7:**
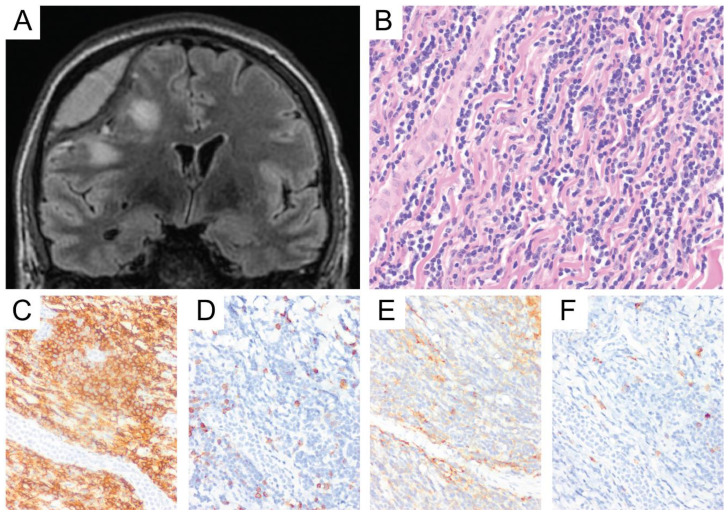
MALT lymphoma of the dura. This lymphoma typically presents as an enhancing dural-based mass (so-called “dural tail”), often mistaken for meningioma (**A**, coronal section, magnetic resonance imaging FLAIR sequence). The tumor cells are small-to-medium-sized with irregular nuclei and abundant cytoplasm and are embedded within a background of fibrous dura (**B**, original magnification ×400). The tumor cells are often positive for CD20 (**C**) and negative for CD5 (**D**), CD10 (**E**), and CD23 (**F**) (**B**–**F**, original magnification ×200).

Dural MZL is a rare extra-axial and intradural lymphoma that shows no direct extension into the brain parenchyma. Dural MZL is almost always localized and systemic involvement is uncommon, although complete staging is still warranted for treatment planning. Clinically, this entity is more common in women [75]. Its presentation is usually insidious, and the symptomatology is dependent on the location of the tumor and may include seizure, headaches, and focal sensory and/or motor changes. Imaging typically shows an extra-axial-enhancing localized mass with associated thickening and enhancement of the adjacent dura (“dural tail” appearance; Figure 7A), mimicking meningioma in many cases, as well as subdural hematoma. The disease typically follows an indolent course, and in cases of localized disease, surgical resection is often sufficient without the need for adjuvant therapy [76]. Low-dose radiation therapy has also been applied with efficacy [77,78]. In a recent retrospective study with a median follow-up of 64 months, all patients were alive with a 3-year progressive-free survival of 89% [78].

Histologic sections typically reveal a dense low-grade lymphoid neoplasm within dura, which can occasionally colonize follicles. The neoplastic lymphocytes are usually small-to-medium-sized and contain irregular nuclei with moderate to abundant pale cytoplasm (Figure 7B). Morphology can be variable and include plasmacytoid/plasmacytic and monocytoid morphologies, although occasional larger cells can be present [78,79]. Dutcher bodies and Mott cells can be seen in some cases [78,79]. Dural MZL (especially those with a prominent plasmacytic component) shows light chain-restriction and may be associated with tumefactive amyloid deposition [75]. The lymphoma cells have a similar immunophenotype as other marginal zone lymphomas with positivity for CD20 and negativity for CD5, CD10, and CD23 in most cases (Figure 7C–F) by immunohistochemistry or flow cytometry. The lymphoma cells often show expression of Bcl-2 and ~50% cases can be positive for CD43 [78]. The proliferative indices are often low (less than 5–10%) [78]. Like MALT/marginal zone lymphomas in other sites, a subset of primary dural MZL cases may show positive staining for IgG4 in the absence of an IgG4-related disorder [73,79]. A molecular profiling study of 14 cases of dural MZL revealed recurrent alterations that correlated with different morphologic patterns. For example, those with plasmacytic differentiation harbored frequent *TNFAIP3* inactivating mutations, whereas dural MZL with variable monocytoid morphology had activating *NOTCH2* and co-occurring *TBL1XR1* mutations [73]. The mutational profile of dural MZL thus appears distinct from parenchymal-based PCNSL, which are predominantly DLBCL. The vast majority of dural MZL cases have shown clonal *IGH* gene rearrangement [73,79]. Trisomy 3 is another recurrent genetic abnormality per multiple studies [73,75]. Additional studies correlating molecular status with clinical outcomes are currently lacking.

## 11. Peripheral T-Cell Lymphoma, not Otherwise Specified (PTCL, NOS) and Anaplastic Large Cell Lymphoma (ALCL)

The frequency of primary CNS T-cell lymphomas (PCNSTCL) seems to be slightly higher in East Asia in comparison to in Western countries, with a reported frequency of 7.4–8.5% and 3.5%, respectively [80,81]. Shenkier et al. reported the largest series to date that included 45 patients with primary CNS T-cell lymphomas [82]. The median age of presentation was 59.5 years (range 3–84 years) [82]. All patients showed no systemic involvement and their lymphomas were confined to the brain (42/45, 93%), spinal cord (2/45, 4%), and meninges (1/45, 2%) [82]. Like PCNS DLBCL, most patients presented with supratentorial lesions [82]. The most common sites of involvement in decreasing frequency included the cerebral hemisphere, basal ganglia, corpus callosum, brainstem, and cerebellum [82]. Villegas et al. reported posterior fossa involvement in 54% of the patients in their series [83]. PCNSTCL, in comparison to PCNS DLBCL, showed a greater male preponderance, a higher frequency of B symptoms at the initial presentation, and a lower incidence of ocular involvement [82]. Involvement of deep brain structures (basal ganglia, corpus callosum, brainstem, and/or cerebellum) and multiple lesions at diagnosis were seen in, respectively, 30–36% and 29–56% of patients [82,83]. A range of clinical manifestations have been reported, including headache, aphasia, facial paralysis, facial and upper limb sensory abnormalities, speech abnormalities, ataxia, leg weakness, and difficulties in short-term memory [83].

Peripheral T-cell lymphoma, not otherwise specified (PTCL, NOS) constitutes the majority of PCNSTCL, comprising 83% (15/18) of the Menon’s series, followed by anaplastic large cell lymphoma (ALCL) [84]. In Shenkier’s series, pathology reports were available in 25 out of 45 patients, and the lymphoma cells were described as small to medium in 12 cases (50%) and “pleomorphic” or compositions of “medium to large” cells in the remaining cases, with three of the previous showing features of ALCL [82]. In comparison to PCNS DLBCL, establishing a diagnosis of PTCL, NOS is more challenging because T-cell-predominant infiltrate can also be seen in reactive condition (i.e., in the setting of other CNS neoplasms or inflammatory/infectious conditions). The diagnosis of PTCL, NOS is based on the combination of histomorphology, immunophenotype, and T-cell receptor gene rearrangement study, emphasizing the importance of obtaining an adequate diagnostic specimen and optimal specimen allocation for different ancillary studies in the fresh state.

**Figure 8:**
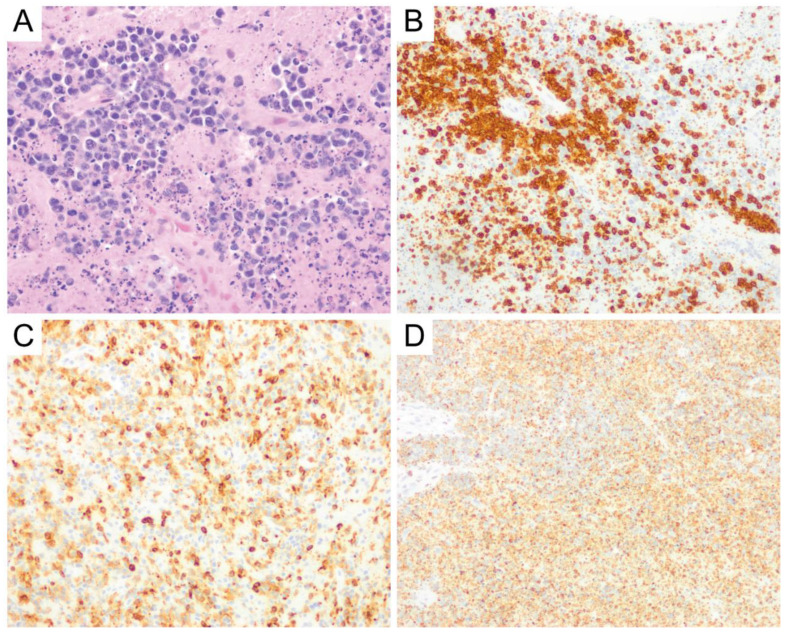
Primary CNS peripheral T-cell lymphoma, not otherwise specified (PTCL, NOS). This example shows enlarged lymphoid cells with convoluted nuclei with scant to moderate cytoplasm embedded within swaths of associated necrosis (**A**, original magnification ×400). Tumor cells were immunoreactive for CD3 (**B**) along with other cytotoxic T-cell markers such as CD8 (**C**) and TIA1 (**D**) (**B**,**C**, original magnification ×200). They were negative for NK-cell markers and EBV ISH, arguing against an NK/T-cell lymphoma. These tumor cells also showed no expression of B-cell markers, arguing against a B-cell neoplasm. This example had T-cell receptor (TCR) beta and gamma gene rearrangements. The majority of the neoplastic cells showed weak cytoplasmic CD30 immunostaining, arguing against ALCL.

The lymphoma cells in the primary CNS PTCL, NOS are mostly small-to-medium-sized with dense, hyperchromatic nuclei, irregular nuclear contour, occasional distinct nucleoli, and scant cytoplasm [84]. Medium to large cells or predominantly large cells can also be seen in a small portion of the cases [84] (Figure 8A). Tumor cells showed perivascular arrangement (angiocentricity) in 67% of Menon’s series [84]. Expansion of the Virchow–Robin space by lymphoma cells was also seen in the latter [84]. Necrosis is frequently seen, as well as background gliosis and histiocytes [84]. The presence of mixed inflammatory cells, i.e., lymphocytes, plasma cells, neutrophils, and eosinophils, favors an inflammatory process [84], but it can be seen in T-cell lymphomas as well.

By immunohistochemistry, almost all primary CNS PTCL, NOS expressed CD3 [84] (Figure 8B). The lymphoma cells more commonly showed a cytotoxic phenotype with positive expression of CD8 (Figure 8C), TIA1 (Figure 8D), granzyme B, and perforin [84]. CD4 expression was seen in 33% of Menon’s series and one case showed a mix expression of CD4 and CD8 [84]. Partial and total loss of expression of CD5 was frequently seen (60%), followed by partial/total loss of expression of CD7 and CD2 [84]. The expression of CD56 was not reported, and rare EBV-positive cells on EBER ISH and LMP1 immunohistochemical stain were observed in a small number of cases [84]. Although CD30 expression can be seen in PTCL, NOS, it is usually of a variable intensity [12]. A diffuse and strong expression of CD30 in all lymphoma cells should prompt a consideration for anaplastic large cell lymphoma (ALCL). The Ki-67 proliferation index was more than 50% [84]. Most cases of primary CNS PTCL, NOS seem to be derived from αβ-T-cells, as reflected by expression of βF1 immunohistochemical stain on the lymphoma cells [84]. T-cell gene rearrangement identified a clonal rearrangement pattern in 12/15 of PTCL, NOS in Menon’s series, one restricted pattern, one suspicious for a significant clonal rearrangement, and one case with insufficient DNA [84]. Menon et al. also performed next-generation sequencing mutation panel targeting mutation hotspots in genes previously reported to be mutated in T-cell lymphomas and in genes involved in T-cell signaling pathways in 10 out of 15 of their PTCL, NOS cases. The authors found no common mutation in multiple cases, suggesting molecular heterogeneity [84].

**Figure 9:**
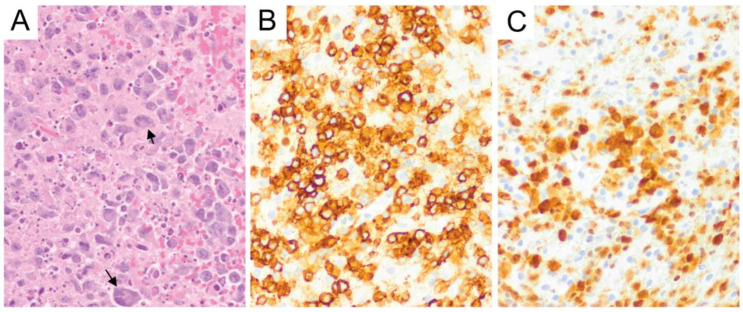
Anaplastic large cell lymphoma. Anaplastic large cell lymphoma is composed of large lymphocytes with frequent “hallmark” cells (arrows), which appear as multinucleated giant cells and Reed-Sternberg-like cells (**A**, original magnification ×400). This tumor is immunoreactive for CD30 (**B**), showing membranous and Golgi/perinuclear dot-like staining patterns, and can further be stratified by ALK status (**C**) (**B**,**C**, original magnification ×400).

The second most common subtype of primary CNS T-cell lymphoma is anaplastic large cell lymphoma (ALCL). Leptomeningeal involvement can be seen in ALCL and grossly manifests as tan-white nodules on the dural surface [84]. The ALCLs in Menon’s series showed cohesive aggregates/sheets in two cases and were scattered throughout the white matter in one case [84]. The lymphoma cells show classic features of ALCL, such as large cells with vesicular chromatin, distinct nucleoli, and abundant cytoplasm with frequent “hallmark” cells [84] (Figure 9A). The distinction between ALCL and PTCL, NOS is based on the diffuse and strong expression of CD30 in the former [12] (Figure 9B). In systemic ALCL, ALK-positive cases generally have a more favorable prognosis than ALK-negative cases [12]. It is uncertain whether the previous holds true in PCNS ALCL. However, ALK immunohistochemical stain should be performed in all ALCL cases when possible (Figure 9C).

## 12. Conclusions

PCNSL is a rare entity, and while the majority of cases are diffuse large B-cell lymphoma, other entities can pose a challenge in diagnostics. Adequate and proper sample collection (i.e., before corticosteroid treatment) and familiarity with different types of lymphomas that arise in the CNS is of utmost importance to guide a proper allocation of specimen for various ancillary studies.
